# In vivo dynamics of pro-inflammatory factors, mucins, and polymorph nuclear neutrophils in the bovine oviduct during the follicular and luteal phase

**DOI:** 10.1038/s41598-023-49151-9

**Published:** 2023-12-15

**Authors:** L. Neubrand, H. Pothmann, U. Besenfelder, V. Havlicek, C. Gabler, M. Dolezal, C. Aurich, M. Drillich, K. Wagener

**Affiliations:** 1 Clinical Unit for Herd Health Management in Ruminants, University Clinic for Ruminants, Department for Farm Animals and Veterinary Public Health, University of Veterinary Medicine Vienna, Vienna, Austria; 2 Reproduction Centre Wieselburg RCW, Institute for Animal Breeding and Genetics, Department for Biomedical Sciences, University of Veterinary Medicine Vienna, Vienna, Austria; 3 Institute of Biotechnology in Animal Production, Interuniversity Department of Agrobiotechnology (IFA Tulln), University of Natural Resources and Applied Life Sciences Vienna, Tulln, Austria; 4 Institute of Veterinary Biochemistry, Faculty of Veterinary Medicine, Freie Universität Berlin, Berlin, Germany; 5 Platform for Bioinformatics and Biostatistics, Department for Biomedical Sciences, University of Veterinary Medicine Vienna, Vienna, Austria; 6 Centre for Artificial Insemination and Embryo Transfer, Department for Small Animals and Horses, University of Veterinary Medicine Vienna, Vienna, Austria; 7 Present Address: Unit for Reproduction Medicine and Udder Health, Clinic for Farm Animals, Faculty of Veterinary Medicine, Freie Universität Berlin, Berlin, Germany

**Keywords:** Molecular biology, Physiology

## Abstract

Dynamic functional changes in the oviductal microenvironment are the prerequisite for the establishment of pregnancy. The objective of this study was to gain the first insights into oestrous cycle-dependent dynamics of polymorph nuclear neutrophils (PMN) and the mRNA abundance of selected genes and their correlations in the oviduct of living cows. Mini-cytobrush samples were taken from the oviducts of healthy heifers (n = 6) and cows (n = 7) during the follicular (FOL) and luteal phase (LUT) by transvaginal endoscopy. Total RNA was isolated from the samples and subjected to reverse transcription-quantitative PCR for selected pro-inflammatory factors, glycoproteins, and a metabolic marker. The percentage of PMN was determined by cytological examination. The mean PMN percentage was 2.8-fold greater during LUT than FOL. During LUT, significantly greater mRNA abundance of the pro-inflammatory factors *IL1B, CXCL1, CXCL3*, and *CXCL8* was observed. The *OVGP1* mRNA abundance was twice as high during FOL than in LUT. Pearson correlation, principal component analysis and heatmap analyses indicated characteristic functional patterns with strong correlations among investigated factors. Using this novel approach, we illustrate complex physiological dynamics and interactions of the mRNA expression of pro-inflammatory factors, mucins, *OVGP1*, and PMN in the oviduct during the oestrous cycle.

## Introduction

Dynamic physiological adaptations of the oviductal microenvironment within the oestrous cycle are crucial for fertility in cattle and other species^1–3^. In the dairy industry, fertility is a fundamental prerequisite for economic efficiency. Embryonic losses occur with a high incidence at early stages of pregnancy. In high-producing dairy cows, embryonic losses become more apparent especially before day 8 after fertilization^4^. Hence, there is a high probability that developmental deficiencies originate from the oviduct. The strictly time-coordinated and oestrous cycle-dependent dynamics of oviductal adaptations are linked to a sequence of key reproductive events, such as gamete transport, sperm capacitation, oocyte fertilization and cleavage, and early embryo development^5^.

In general, reproductive function is strongly related to the endocrine system^6,7^. Functional and morphological changes in the genital tract are regulated by hormonal shifts during the different stages of the oestrous cycle^8^. At the molecular level, changes in the mRNA expression of maternal epithelial cells exist during the oestrous cycle both in the uterus^9^ and oviduct^10^. For example, the secretion of the oviduct-specific glycoprotein 1 (OVGP1) by bovine oviductal epithelial cells (BOEC) depends on the concentration of oestrogens, which result in greater OVGP1 secretion during the follicular phase compared to the luteal phase in many species, as reviewed by Buhi et al.^11^. The OVGP1 is the major oviductal glycoprotein in many species^11^, and in the bovine oviduct, OVGP1 is important for sperm viability, fertilization, and embryo development^11,12^.

Physiologically, inflammatory processes are constantly required in the reproductive tract, for example in response to the introduction of semen or bacteria during mating or insemination or at parturition, or internally by the arrival of the developing embryo or foetus. The expression of immune-related genes in the reproductive tract ensures that it can withstand the inevitable mechanical, bacterial, and functional penetration that occur after calving^9,13^ and is controlled by the endocrine system^9^.

The metabolic, endocrine and inflammatory mechanisms are highly interlinked and the dynamics of the oviductal micromilieu are impacted by local and systemic processes. Therefore, it essential to study the oviduct under in vivo conditions. Most studies comparing oviductal conditions between different stages of the oestrous cycle, however, have been performed on slaughtered animals^10,14,15^ or in vitro^13^, mainly due to the difficulty accessing the oviduct for in vivo sampling. In the past 20 years, transvaginal endoscopy (TVE) has been established as a technique that is used to gain access into the oviduct to study the oviductal microenvironment in living animals^10,16,17^. The fact that in vivo culture in the oviduct leads to embryos with greater quality and stability than in vitro culture^18,19^ highlights the high level of complexity of the oviductal microenvironment. Information about the complex oviductal physiology will contribute to a better understanding of the circumstances that are related to subfertility.

In our study we used TVE-guided intra-oviductal sampling with a mini-cytobrush (mCB) to gain the first insights into the in vivo dynamics of the oviductal microenvironment by analysing the presence of polymorph nuclear neutrophils (PMN) and the mRNA abundance of selected genes, which are known to be related to inflammation and fertility. We hypothesized that the mRNA abundance of pro-inflammatory factors and mucins and the PMN percentage in the oviduct differ between the follicular (FOL) and luteal phase (LUT) of the oestrous cycle.

## Results

### Oviduct samples and PMN percentage

**Figure 1:**
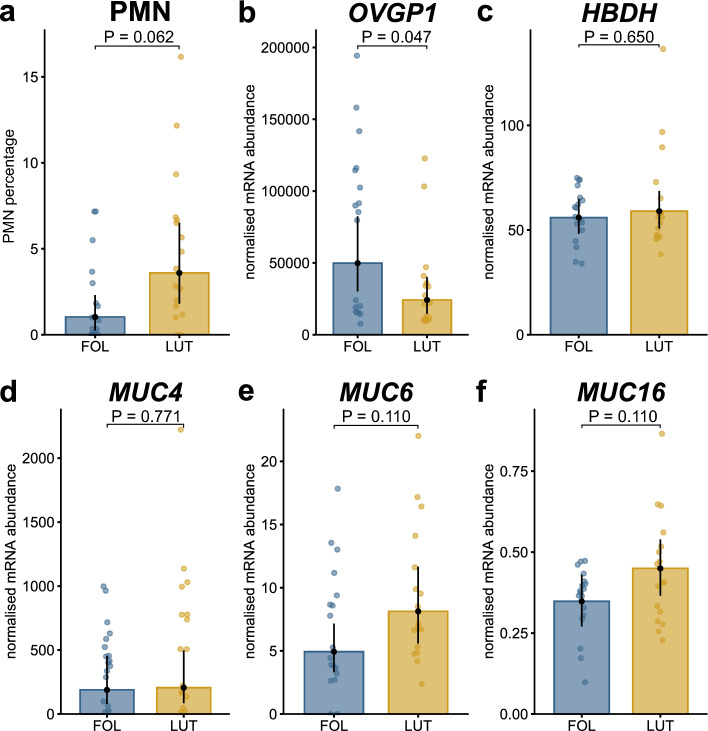
Bar plots based on back-transformed estimated marginal means. The difference of the mean percentage of polymorph nuclear neutrophils (PMN) between the follicular (FOL) and the luteal phase (LUT) was close to significance (**a**). The mRNA abundance of the oviduct-specific glycoprotein 1 (*OVGP1*) differed significantly between both stages of the oestrus cycle (**b**), whereas for hydroxybutyrate dehydrogenase (*HBDH*), and the mucins MUC4, 6 and 16 no differences were found (**d**–**f**). Multiple testing corrected p-values are given on the line above boxes. Note different scales on y-axis.

Sampling of both oviducts in heifers (four sampling sessions) and cows (two sampling sessions) resulted in 60 samples. Table 1 shows each single oviductal sample with the presence (n = 46) and absence (n = 14) of PMN and indicates the non-sampled oviducts. Sampling was successful in 81.8% (39/48) and 75.0% (21/28) of the cases in heifers and cows, respectively. The reason for unsuccessful sampling was continuous restlessness of the animals during sampling or the presence of ovaries that were too small to allow for appropriate fixation of the oviduct by rectal manipulation. We detected PMN in 76.7% (n_positive_ = 46, n_negative_ = 14) of all samples (n_total_ = 60). The cytological examination revealed greater numerically estimated marginal means of the PMN percentage during LUT (4.9%, confidence interval (CI) 2.9–6.9%) than during FOL (1.8%, CI − 0.2 to 3.7%), which was close to significance (p = 0.062, Fig. 1a).

**Table 1:** Single oviductal samples with the presence (+ , n = 46) and absence (− , n = 14) of polymorph nuclear neutrophils (PMN).

Animal	Follicular phase		Luteal phase		Follicular phase		Luteal phase	
	d0		d10		d21		d31	
	Ipsi	Contra	Ipsi	Contra	Ipsi	Contra	Ipsi	Contra
H_1_	+	−	+	+				
H_2_	+	+	+	+	+	+	+	+
H_3_	−	+	+	+	+	+	+	+
H_4_	−		+		+	+	+	
H_5_	−	+	+	+	+	+	+	+
H_6_	+	+	−	+	+	+		
C_1_	−		−					
C_2_	+	−	+					
C_3_	+	−	−	+				
C_4_	−		+					
C_5_	−	+	+					
C_6_	+	+	+					
C_7_	−	−	+	+				

Non-sampled oviducts are presented as blank spaces (n = 16). Samples were taken from six heifers (H_1-6_) and seven cows (C_1-7_) on consecutive days (d) of the oestrous cycle. Cows were sampled at d0 (follicular phase) and d10 (luteal phase). Heifers were additionally sampled at d21 (follicular phase) and d31 (luteal phase). Samples were taken from both oviducts, and which oviduct was ipsi- and contralateral to the dominant follicle during the follicular phase and ipsi- and contralateral to the corpus luteum during the luteal phase is indicated.

### Relative mRNA abundance of pro-inflammatory factors and mucins

The quality of the mRNA of selected samples was evaluated as high (RIN 8.7–9.5).

*OVGP1* was the only factor with a significantly greater mRNA abundance during FOL than during LUT (2.06-fold, p = 0.047, Fig. 1b). The metabolic factor hydroxybutyrate dehydrogenase (*HBDH*) abundance did not differ between both stages of the oestrous cycle (Fig. 1c).

**Figure 2:**
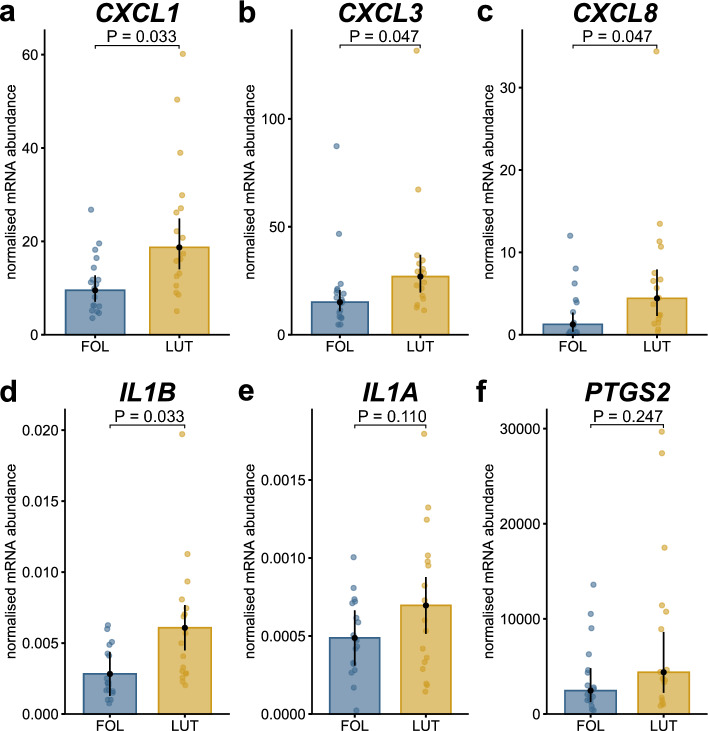
Bar plots based on back-transformed estimated marginal means. The mRNA abundance of chemokine CXL ligand (*CXCL*)1*, CXCL3, CXCL8,* and interleukin (IL)*1B* differed significantly between the follicular (FOL) and the luteal phase (LUT) (**a**–**d**). For *IL1A* (**e**) and prostaglandin-endoperoxide synthase 2 (*PTGS2*) (**f**), no significant differences were found. Multiple testing corrected p-values are given on the line above boxes. Note different scales on y-axis.

The pro-inflammatory factors *CXCL1* (2.0-fold)*, CXCL3* (1.8-fold), *CXCL8* (3.5-fold) and *IL1B* (2.2-fold) were significantly greater expressed during LUT than during FOL (Fig. 2a–d). No significant differences were found for *ILA* and prostaglandin-endoperoxide synthase 2 (*PTGS2*) (Fig. 2e,f).

Similar to the pro-inflammatory factors, the abundance of the mucins *MUC4*, *MUC6*, and *MUC16* was greater during LUT than FOL but the difference was not significant (p > 0.05 for all factors) (Fig. 1d–f).

### Heatmap and principal component analysis representing functional clustering of mRNA abundance, PMN percentage and progesterone

**Figure 3:**
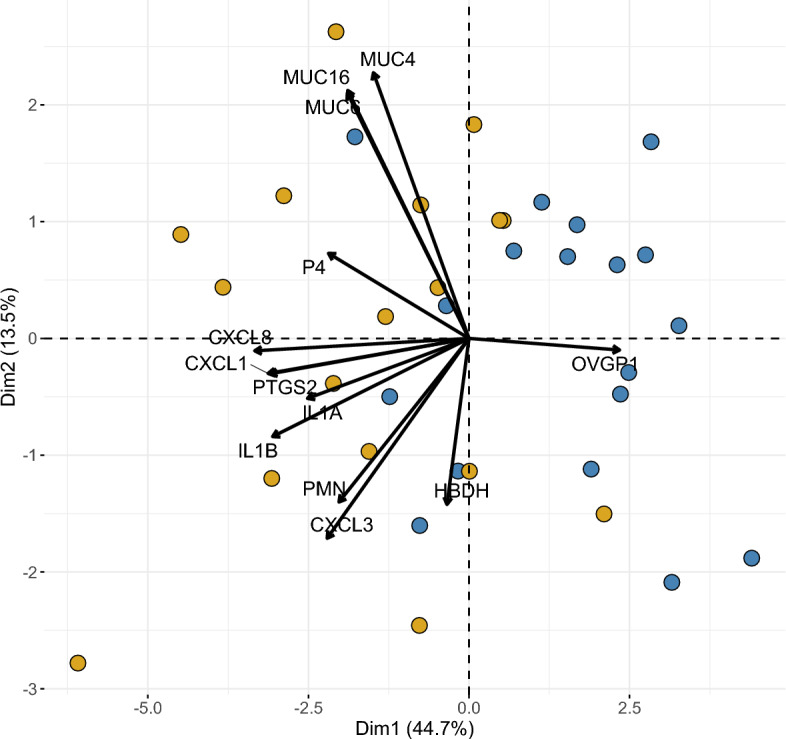
Principal Component Analysis (PCA) of residuals. The PCA displays scores of samples (dots) collected during the follicular (blue) and the luteal phase (orange) and loadings of each variable (vectors). A small angle between vectors indicates a positive correlation between phenotypes, 90° indicates no correlation and 180° a negative correlation.

Principal component analysis (PCA) illustrates clusters within analysed factors (Fig. 3). The first dimension of the PCA showed a non-perfect separation of the data points according to the two stages of the oestrous cycle (blue = FOL; yellow = LUT) and accounted for almost half (44.7%) of the variation. Dimension 2 accounted for 13.5% of the variation. The mucins and progesterone (P4) clustered together in the upper left quadrant, while the pro-inflammatory factors and PMN formed a cluster in the lower left quadrant. The orientation of the *OVGP1* vector showed that *OVGP1* abundance is diametral to all observed phenotypes. The *HBDH* vector was close to 90° to *OVGP1*, indicating no correlation between these two factors.

**Figure 4:**
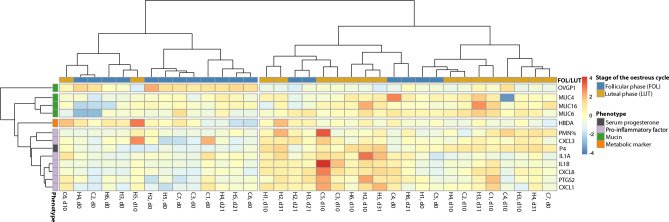
Heatmap for hierarchical clustering based on correlation coefficient distances. Rows represent selected phenotypes (light purple: pro-inflammatory factors, grey: serum progesterone concentration, green; glycoproteins, orange: metabolic marker. The columns represent single samples collected during the follicular (blue) and the luteal phase (orange).

The results of the heatmap analysis were analogous to PCA and illustrate clustering of the phenotypes according to their biological functions (Fig. 4). *OVGP1* built its own branch and clustered furthest from all other phenotypes. *OVGP1* showed increased relative abundance (red) during FOL and decreased relative abundance (blue) during LUT. Mucins and pro-inflammatory factors formed two distinct clusters. The columns on the left mainly represent samples taken during FOL, and show downregulated mRNA abundance of mucins and pro-inflammatory factors, while the columns on the right, after the first split, show mainly upregulated mRNA abundance. P4 clustered among the pro-inflammatory markers and showed similar trends in up- and downregulation. *HBDH* formed a single branch most closely related to the pro-inflammatory factors.

### Correlations among mRNA abundance, PMN percentage and P4

**Figure 5:**
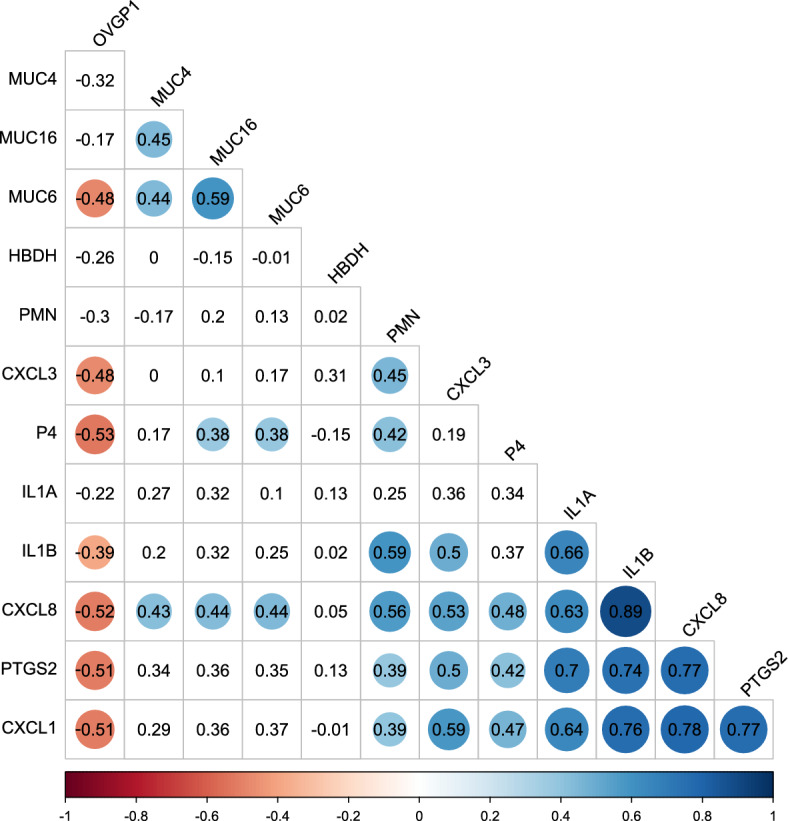
Correlations between the abundance of genes, PMN and P4 levels. Pearson correlation coefficients are indicated and illustrated as dots for either positive (blue) or negative significant correlations (red) (Multiple testing corrected p < 0.05). The size and colour-intensity of dots symbolize the strength of correlation. Numbers without dots represent no significant correlation.

Figure 5 shows correlations between blood P4 levels, percentage of PMN and mRNA gene abundance. All pro-inflammatory factors and PMN showed a similar functional pattern as they were positively correlated with P4. Significant correlations were found between P4 and PMN, *CXCL8*, *CXCL1* and *PTGS2*, with *r* ranging between 0.42 and 0.48 (p < 0.05 for all correlations). On the contrary, *OVGP1* was negatively correlated with P4 (*r* = − 0.53; p = 0.004). Among the selected mucins, *MUC6* and MUC16 were positively correlated with the concentration of P4 (both *r* = 0.38; p < 0.05). Similar to this result, all pro-inflammatory factors were positively correlated with PMN as well, with the strongest correlation between PMN and *IL1B* (*r* = 0.59; p < 0.001) and *CXCL8* (*r* = 0.56; p = 0.002). The strongest correlation among pro-inflammatory factors was found between *CXCL8* and *IL1B* (*r* = 0.89; p < 0.001) and between *CXCL8* and *CXCL1* (*r* = 0.78; p < 0.001).

## Discussion

The functional adjustments within the oestrous cycle prepare the reproductive tract for creating a compatible environment to match the different needs of gametes and the developing early embryo^1,3,5^. Whereas the uterine environment has been well described, less is known about oestrous cycle-dependent dynamics in the oviduct. The presence of steroid hormone receptors in the oviduct and other previous in vitro findings indicate that the ovarian oestrous cycle also controls the oviductal environment^8,10^. To the best of our knowledge, we provide the first insights into the dynamics of oestrous cycle-dependent functional changes in the oviduct in vivo by using TVE. This minimally invasive technique facilitated multiple sampling of the same animal within a short period of time.

The fertility of animals strongly depends on a finely tuned immune response during sequential events, such as the exposure to microorganisms by the opened cervix during oestrus and calving, and while introducing allogenic semen or hosting a semi-allogenic embryo, as reviewed by Talukder et al.^20^. A balanced activation and function of PMN are crucial for sustainable reproductive health and fertility. Subclinical endometritis is caused by persistent endometrial inflammation, which is accompanied by PMN infiltration into the uterus^21,22^. Despite clinical recovery, endometrial inflammation compromises the fertility of dairy cows in the long term. Owhor et al.^23^ showed that endometritis is often accompanied by salpingitis of both oviducts. On the other hand, a certain inflammatory response at the cellular and molecular level was favourable for insemination success^24,25^. Our findings suggest the involvement of PMN in the maintenance of physiological conditions in the oviduct, since PMN were present in most samples and the samples originated from healthy animals. As we even detected PMN in the untouched oviduct of heifers, our results illustrate the physiological involvement of these immune cells in the oviduct, not necessarily being activated by foreign material, such as bacteria or sperm. This is in accordance with a previous study illustrating the presence of PMN in the bovine oviduct of slaughtered animals under physiological conditions during oestrus and di-oestrus^26,27^. The latter studies, however, did not provide any information about oestrous cycle-dependent PMN dynamics in the ampullary region of the oviduct. In our study, the presence of PMN tended to be lower during FOL compared to LUT and we observed a moderate positive correlation between the PMN percentage and P4 concentration (*r* = 0.42). The lower PMN presence during FOL might be important to prevent sperm phagocytosis after insemination. Marey et al.^27^ demonstrated that the phagocytotic function of PMN is suppressed by sperm binding to BOEC and by an increase in the concentration of luteinizing hormone to protect spermatozoa in the isthmic reservoir of the oviduct. In addition, direct sperm-oviduct contact induces an anti-inflammatory immune response and further decreases the phagocytic activity of PMN^28^. Since our animals were not inseminated before sampling, it can be speculated that this anti-inflammatory environment is already established in the oviduct without the presence of sperm to prevent PMN infiltration. The balanced regulation of PMN presence and function can be considered as crucial for maintaining physiological conditions in the oviduct. Since uterine PMN and the animal’s fertility were not examined in our study, it remains an open question whether PMN might migrate from the uterus into the oviduct and interfere with early embryo development. Investigating the impact of pathological conditions in the uterus, such as clinical or subclinical endometritis, on the presence of PMN in the oviduct in further studies might help to fill the gap in understanding mechanisms that lead to a long-term decline in fertility.

Bauersachs et al.^10^ analysed the bovine oviductal transcriptome in slaughtered animals. In accordance with these results, the abundance of genes encoding for pro-inflammatory factors decreased during FOL and increased during LUT in the hypothesis testing and heatmap analysis. In addition, the concentration of P4 was positively correlated with the abundance of all pro-inflammatory factors and the presence of PMN. Interestingly, contrasting results have been reported for the uterus, where during FOL, pro-inflammatory factors are upregulated compared to LUT^9^. Considering that the immune system of the oviduct responds differently to steroid hormones than that of the uterus, our results assume a precise time-coordinated activation of immunological processes within the entire genital tract. In our mRNA analyses, we focused on specific pro-inflammatory factors that are involved in key events in the bovine reproductive tract^9,14,25,29^. We found characteristic functional patterns related to the chemotaxis of PMN. The strong correlation between *CXCL1, CXCL8* and *IL1B* and the presence of PMN mirrors the synergistic chemotactic effect of *CXCL8*, *IL1B* and *CXCL1* on PMN^30^. Besides the chemo-attractive effect, several studies have related an altered abundance of *CXCL8* and *IL1B* to uterine resistance and susceptibility^29^. Paula-Lopes et al.^31^ reported a beneficial effect of IL1B on oocytes and the embryo during early stages of development, resulting in improved blastocyst rates. Our findings support the assumption that these genes are involved in physiological processes in the oviduct as well. In analysing the effect on fertility in our study, the restricted number of animals was a limiting factor with regard to ensuring representative results. Future studies should investigate the effect of genital tract disorders on the oviductal microenvironment by including a broader set of factors related to the immune response and fertility by using quantitative transcriptomic and proteomic approaches^32,33^.

PTGS2 is an inducible enzyme that stimulates the enzymatic transition of arachidonic acid to precursor PGH2. In the next steps of PG-synthesis, specific enzymes catalyse the conversion into various PGs, inter alia PGF2α and PGE. Both have rather antagonistic functions in the reproductive tract via auto- and paracrine effects^34^. Therefore, previous in vitro studies are difficult to interpret, as the potential influence of adjacent cells might not have been considered. In vitro research has shown that PGs play an important role in sperm survival in the oviduct^27^. Information about PTGES2 in the bovine oviduct of living animals is, however, rare. Previous studies reported interleukin (*IL*)*1A* and *IL1B* induced expression of *PTGS2* in the endometrium of humans, pigs, and cattle^35–37^. We found a strong positive correlation between *PTGS2* and *CXCL8* and *IL1B*, suggesting similar cytokine-regulated PG synthesis in the oviduct to that described for the uterus. The *PTGS2* mRNA abundance did not differ significantly between FOL and LUT, which is consistent with Odau et al.^38^ who found no oestrous cycle-dependent changes in either the mRNA or protein abundance of oviductal *PTGS2* in slaughtered animals. In contrast, in vitro stimulation of BOEC with 17-beta oestradiol resulted in increased PGE_2_ and PGF_2α_ synthesis^39^ and increased *PTGS2* mRNA abundance^38^. It remains under speculation whether the described in vitro effect was too short or too low to be detected in vivo. Nevertheless, our results support the idea of local, cytokine-stimulated PG synthesis in the bovine oviduct and provide a solid basis for future studies on the regulation of PTGS2 by ovarian steroid hormones.

Mucins are membrane-associated or secretory glycoproteins covering the epithelium layer of, e.g., the oviduct and the uterus^11^. Previous studies indicated that in the uterus, the absence of downregulation of mucins interferes with the embryo-maternal contact, contributing to repeated breeding^29,40^. Mucins have been analysed in the bovine cervix and uterus^29,40^ and in the oviduct *in vitro*^14^, but information about their dynamics in the oviduct is leaking. In our study, the stage of the oestrous cycle had no significant effect on MUC4, 6 or 16. A general local response of BOEC to the presence of an embryo has been described by Bauersachs et al.^41^. In addition, MUC secretion was directly upregulated by the human blastocyst^42^. These findings might explain our non-cycle-dependent expression of *MUC*, indicating that *MUC* expression might be generated either directly by the embryo or as a local reaction of BOEC to its presence. Given the relevance of mucins for reproduction, further in vivo studies about their local abundance in response to artificial insemination or to directly transferring embryos into the oviduct might be promising.

OVGP1 (also known as MUC9) can be morphologically assigned to the glycoproteins^11^. Heat-map and PCA analyses revealed that its mRNA abundance was technically differentiated compared to the remaining mucins (*MUC4*, *6*, *16*). This illustrates the special functions of the major oviductal glycoprotein during fertilization and early embryonic development. The oestrogen-dependent expression of *OVGP1* has been identified in several studies either showing a greater abundance during oestrous^43^ or investigating the effect of the administration of oestrogen on prepubertal and ovariectomized animals^44,45^. In the heatmap analysis, *OVGP1* clustered on its own furthest away from all other measured phenotypes, with upregulation during FOL. This is consistent with *OVGP1* being the only phenotype that was significantly upregulated during FOL in the hypothesis testing. In this way, we confirmed prior in vitro results of 17b-estradiol-associated gene expression^46^.

*HBDH* was the only metabolic marker in our study, and its distinct role is reflected by the findings that it showed no significant correlation with the immune-related factors and by its separate clustering in the heat map analysis. The mRNA abundance of *HBDH* did not differ between FOL and LUT, which was not surprizing as it is not regulated by steroid hormones. As HBDH is involved in the metabolism of beta-hydroxybutyrate^47^, it would be interesting to study its contribution to subfertility in cows with subclinical ketosis, which was not the case in our study animals as they had BHB values < 1.2 mmol/L.

Access of the oviduct is a challenge in attempts to study its intricate dynamics. Prior longitudinal in vivo studies have used laparotomy via the flank and had detrimental effects on animal welfare and on the ability to represent the in vivo conditions in the oviduct, especially by interfering with intrinsic inflammatory processes^48^. No increase in the mRNA abundance of pro-inflammatory factors or PMN percentage was observed between the FOL (day 0 and 21) and LUT (day 10 and 31) samples. Therefore, we can largely exclude local inflammation caused by mCB sampling. It is important to note, however, that future in vivo studies with repeated sampling, especially those including fertility outcomes, gamete or embryo development, should be carefully designed to exclude sampling induced inflammation.

Enhanced in vitro models revealed the refined complexity of this organ more deeply and emphasize its involvement in reproduction^10,15,41,49^. Ex vivo studies showed that oviductal function is specifically dispersed throughout its different segments (infundibulum, ampulla, isthmus)^26,50^, which we could not explore by TVE since TVE-guided sampling is limited to the ampullary region. Nevertheless, with our study we were able to demonstrate a great overlap between prior in vitro studies and our in vivo findings and to illustrate the complex physiological mechanisms of the local immune system in the oviduct. It is not possible to predict how far ex vivo techniques conceal true events because of post-mortem changes or the absence of the whole organism, and thus they require a certain in vivo comparison.

## Conclusion

Using TVE we incorporated an innovative tool for repeatedly sampling the oviduct of the same animal under in vivo conditions in a novel manner and gained the first in vivo insights into the complex dynamic regulation of the bovine oviductal microenvironment during the oestrous cycle. Specifically, we showed fluctuations in and interactions between the mRNA of selected pro-inflammatory factors and mucins, PMN and P4. These dynamics manifested in characteristic patterns in their respective functional groups. Hypothesis testing revealed that the mRNA abundance of the pro-inflammatory factors *IL1B*, *CXCL1*, *CXCL3*, and *CXCL8* was significantly greater during LUT than in FOL, whereas the *OVGP1* mRNA abundance was twice as high during FOL than in LUT. Our findings indicate that these genes are involved in physiological processes in the oviduct as well. TVE represents a promising technique for future studies to investigate the physiology of the oviduct and gain a better understanding of early embryonic development, embryo-maternal communication and mechanisms that impede pregnancy.

## Materials and methods

The study was approved by the institutional ethics committee and the national authority according to §8 of the Law for Animal Experiments (Tierversuchsgesetz-TVG; BMBWF GZ-68.205/0014-V/3b/2019). Relevant guidelines and regulations were consistently complied.

### Study animals and inclusion criteria

The study was performed at the teaching and research farm of the University of Veterinary Medicine Vienna (VetFarm Kremesberg). Initially, a total of seven clinically healthy dairy heifers and eleven lactating dairy cows (all Simmental breed) were included in the study. Heifers were sampled from March to November 2016 and cows were sampled from November 2018 to October 2019. Experimental conditions, housing and feeding did not change during the study period. However, to err on the side of caution we fitted a random intercept effect of day of sampling in our hypothesis testing models to account for any environmental changes that could have occurred during the study period.

Heifers were kept in a two-area system with a bedded lying area and a solid feeding alley. Only those heifers whose reproductive tract had not previously been exposed to any interventions were included.

Cows were kept in a group of approximately 80 lactating animals in a free-stall barn with straw-bedded cubicles. In the first and second week postpartum, the blood beta-hydroxybutyrate concentration (BHB) was determined in cows using a handheld meter (Freestyle Precision Neo, Abbott Diabetes Care Ltd., Oxon, UK)^51^. A gynaecological examination was performed between day 21 and 27 and again between day 35 and 41 postpartum by rectal palpation, transrectal sonography (5-MHz linear-array transducer; Easi-Scan, BCF Technology Ltd., Bellshill, Scotland) and vaginoscopy^52^. Only lactating cows without subclinical ketosis, i.e., cows with BHB < 1.2 mmol/L^51^ and cows without clinical endometritis, i.e., cows with clear vaginal discharge and symmetric uterine horns^53^, were enrolled. In addition, none of the cows had ovarian cysts, defined as fluid-filled ovarian structures > 2.5 cm in diameter and without a corpus luteum during the postpartum checks. Neither heifers nor cows received antibiotic or anti-inflammatory treatment prior to sampling.

### Synchronization protocol and exclusion criteria

**Figure 6:**
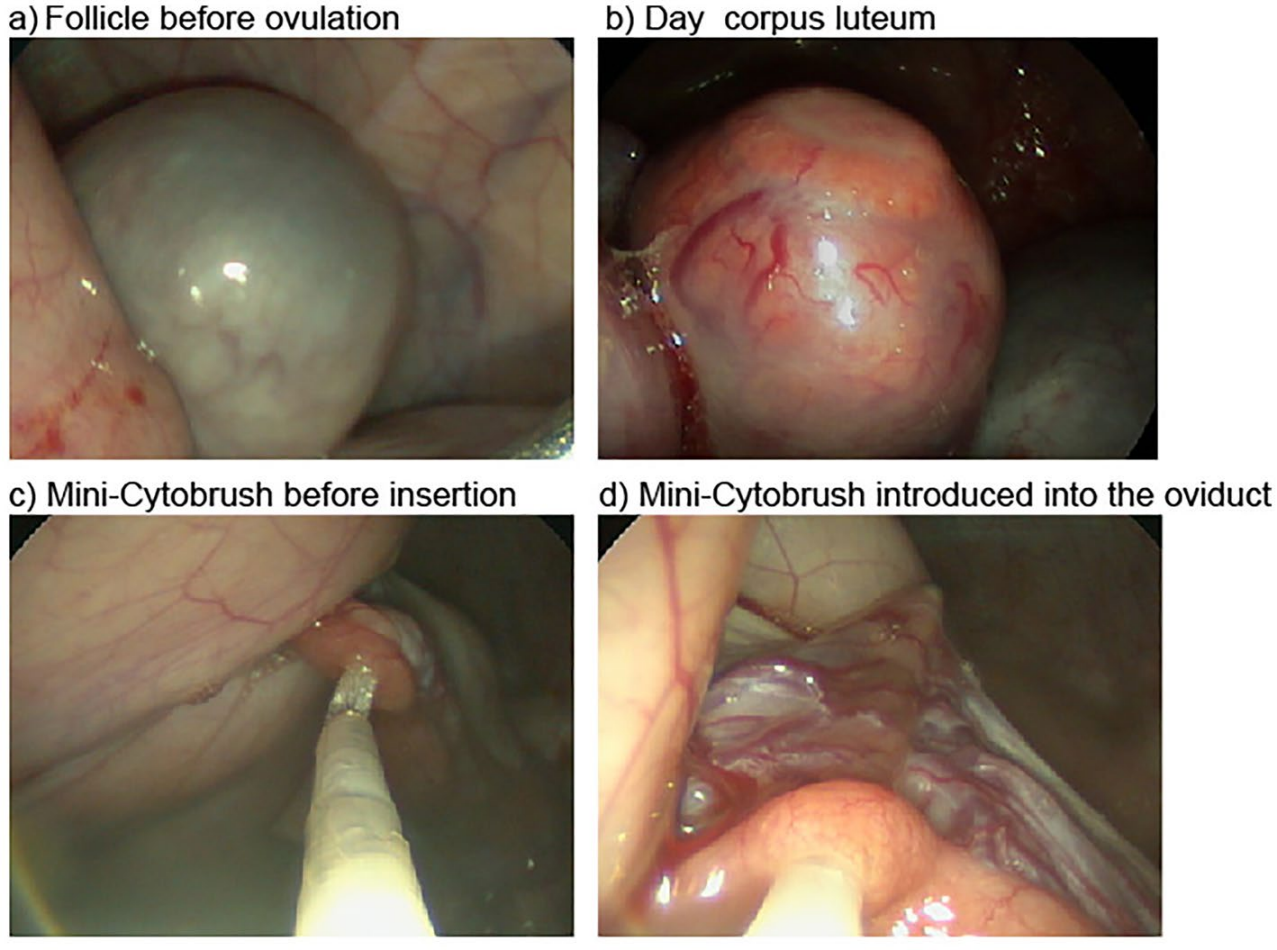
Images of ovarian structures recorded during transvaginal endoscopy. Representative images are shown for the follicular (**a**) and luteal phase (**b**). In addition, the mini-Cytobrush is shown before (**c**) and after insertion (**d**) into the oviduct.

For sampling, cows underwent two sampling sessions within one oestrous cycle, whereas heifers underwent four sessions in two consecutive oestrous cycles. To facilitate terminated sampling, cows and heifers were synchronized using a modified OvSynch protocol as described previously^54^. All animals were sampled on the day of oestrus (the follicular phase, FOL), defined as day 0, and ten days later during the luteal phase (LUT). For heifers, the procedure was also carried out on day 21 (FOL) and day 31 (LUT) after enrolment (Table 2). Efforts were made to sample both the left and right oviducts in each session and these were classified as ipsi- and contralateral to the dominant follicle during the follicular phase, and ipsi- and contralateral to the corpus luteum during the luteal phase. Prior to sampling, the response to oestrus synchronization was assessed during TVA using transrectal sonography (5-MHz linear-array transducer; Easi-Scan, BCF Technology Ltd., Bellshill, Scotland) and also by visual control of the ovaries during TVE. Figure 6 shows representative endoscopic images for the ovarian structures and the sampling procedure with the mCB. One heifer and three cows were excluded due to unsuccessful synchronization of the oestrous cycle, i.e., the absence of a corpus luteum during the luteal phase and a follicle during the follicular phase, or because of ovarian cysts. In addition, a short clinical examination and a gynaecological examination were performed by rectal palpation and vaginoscopy as described above, showing no abnormalities.

**Table 2:** Sampling scheme of the study.

	Follicular phase	Luteal phase	Follicular phase	Luteal phase
	d0	d10	d21	d31
Heifers	x	x	x	x
Cows	x	x		

Samples were taken from heifers (n = 6) and cows (n = 7) on consecutive days (d) of the oestrous cycle. Cows were sampled at d0 (follicular phase) and d10 (luteal phase). Heifers were additionally sampled at d21 (follicular phase) and d31 (luteal phase). Samples were taken from both oviducts.

One cow was excluded because mRNA extraction was insufficient and undetectable with qPCR. This resulted in seven cows and six heifers for the final analyses. Heifers were aged between 14 and 16 months. Four cows were in their first lactation, one in their second, and two cows had had more than two lactations.

### Oviductal sampling

**Figure 7:**
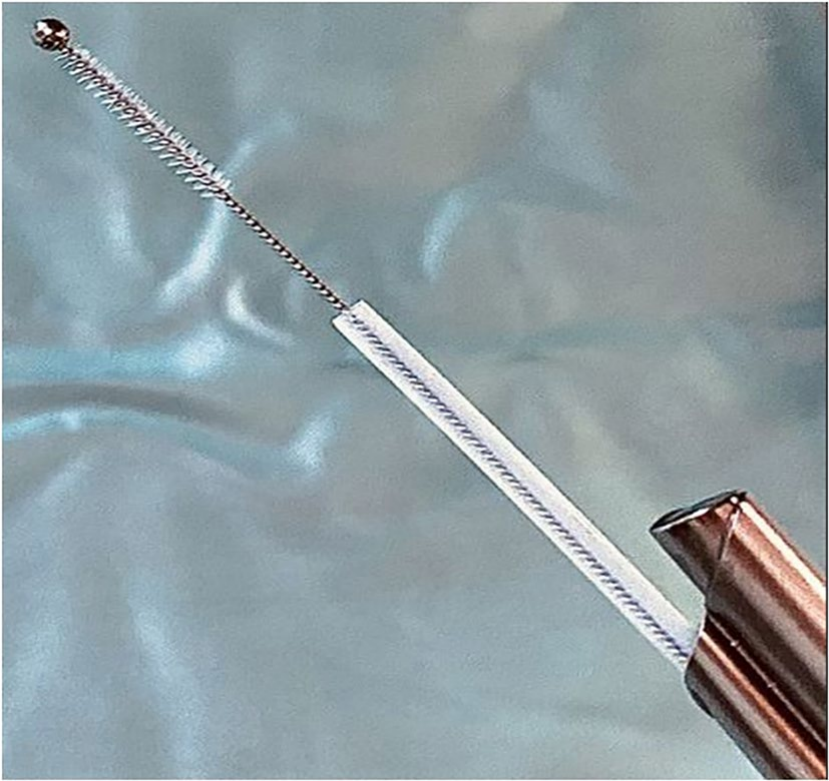
Mini-cytobrush samples were taken from the oviducts by transvaginal endoscopy.

During sampling, cows were fixed in an examination stand. First, the uterus and ovaries were examined by transrectal palpation and sonography to assess uterine health and determine the stage of the oestrous cycle. Prior to oviductal sampling, animals received epidural anaesthesia (3–5 mL 2% procaine hydrochloride, Procamidor®, Richter-Pharma AG, Wels, Austria) and blood samples were collected from the tail vein with VACUETTE tubes (Greiner bio-one, Kremsmünster, Austria). The TVE was performed as described previously^17^. In brief, a universal tube was introduced with a traumatic trocar through the vaginal wall into the peritoneal cavity. Then the trocar was replaced by a tubing system bearing the endoscope (5.5 mm, forward Hopkins optic, Storz, Vienna, Austria) and a working channel with the mini-Cytobrush (mCB) (Fig. 7). The mCB itself was covered by a flexible plastic catheter. Just before sampling, the mCB was pushed forward out of the plastic catheter and inserted through the infundibulum into the ampulla of the oviduct to obtain oviductal epithelial cells. After sampling, the mCB was pulled back into the plastic catheter and the working channel and immediately transferred in Eppendorf tubes and stored at – 80 °C.

### Hormone analysis and cytology

Blood samples were centrifuged at 1800 g for 5 min at room temperature. Plasma was pipetted into 2 ml Eppendorf tubes and stored until further analysis at − 20 °C. Progesterone concentrations were measured using a validated enzyme-linked immunosorbent assay (Progesterone ELISA kit, Enzo Life Sciences, NY, USA) as described by Pothmann et al.^55^. Inter- and intra-assay coefficients of variation were 5.6 and 4.9 ng/mL, respectively.

The mCBs were rolled on disinfected microscope slides for cytological examination. After staining (Hemacolor Rapid staining; Merck, Darmstadt, Germany), 300 cells (epithelial cells and PMN) were counted at 400 × magnification to determine the percentage of PMN in each sample.

### RNA extraction and reverse transcription-quantitative PCR

RNA was isolated according to the manufacturer's protocol of the RNeasy Plus Mini Kit (Qiagen, Hilden, Germany). A slight modification was used for separation of the sampling material from the cytobrush to obtain the maximum amount of cells and thus RNA, as described previously^29^.

The total RNA concentration was measured by spectrophotometry (NanoDrop ND-1000, Peqlab Biotechnology, Erlangen, Germany) at a wavelength of 260 nm. Electrophoresis (Bioanalyzer 2100 expert Version 2.6, Agilent Technologies, Waldbronn, Germany) was performed to measure the RNA Integrity Number (RIN) for quality control. The procedure was in accordance with the manufacturer's protocol for the Eucaryote total RNA Nano assay class. To remove genomic contamination, a digestion mix with DNAses was added^29^. Then, reverse transcription was performed with 500 ng total RNA, 4 μL dNTPs (Bioline, Luckenwalde, Germany), 1.5 μL hexamers, 6 μL reverse transcription buffer, 1 μL MMLV (all Thermo Scientific, Langenselbold, Germany) in a total volume of 60 μL per sample as described by Odau et al.^38^. The qPCR was performed using the StepOne Plus (Applied Biosystems GmbH, Darmstadt, Germany) with a 9 μL reaction mix per sample consisting of 0.2 μL of each primer, 5 μL Sensi fast syber Hi-Rox Kit (Bioline, Luckenwalde, Germany), 3.6 μL water and either 1 μL of the sample premix, 1 μL of the dilution series starting from 1:1000, or 1 μL of water, serving as negative controls. The qPCR was started with a holding stage for denaturing for 2 min at 90 °C. The cycle stage started with another denaturation for 15 s at 90 °C, continued with annealing for 20 s at predefined, gene-specific temperatures (Table 3) according to the protocol, and finished with an elongation phase at 72 °C for 30 s. Melting curve stage for continuous fluorescence measurement started with 15 s at 90 °C following 1 min at 60 °C and another 15 s at 90 °C.

**Table 3:** Primer pairs of selected gene transcripts used for reverse transcription-quantitative PCR.

Gene	Primer sequence	Reference/accession number	Fragment size (bp)	Annealing temperature (°C)
*RPL-19*	for 5′-GGC AGG CAT ATG GGT ATA GG- 3′	Danesh Mesgaran et al.^13^	232	60
	rev 5′-CCT TGT CTG CCT TCA GCT TG- 3′			
*SDHA*	for 5′-GGG AGG ACT TCA AGG AGA GG-3′	Danesh Mesgaran et al.^14^	219	60
	rev 5′-CTC CTC AGT AGG AGC GGA TG-3′			
*SUZ12*	for 5'-TTC GTT GGA CAG GAG AGA CC-3'	NM_001205587	286	60
	rev 5'-GTG CAC CAA GGG CAA TGT AG-3'			
*UXT*	for 5′ –CGC TAC GAG GCT TTC ATC TC- 3′	NM_001037471.2	207	61
	rev 5′ –TGA AGT GTC TGG GAC CAC TG- 3′			
*HBDH*	for 5′ -ATG TCC TCT GTG GCT TCC AG- 3′	NM_001034488.2	347	59
	rev 5′ -CAC AAA CTC CAG CCT CCA TC- 3′			
*OVGP1*	for 5′ -GGG AAA GGT TCG TCA GTT CA- 3′	Danesh Mesgaran et al.^14^	240	60
	rev 5′ -CAT ACG CTT TCT GGA CGA CA- 3′			
*IL1A*	for 5'-TCA TCC ACC AGG AAT GCA TC-3'	Danesh Mesgaran et al.^13^	173	53
	rev 5'-AGC CAT GCT TTT CCC AGA AG-3'			
*IL1B*	for 5′-CAA GGA GAG GAA AGA GAC A- 3′	Danesh Mesgaran et al.^13^	236	53
	rev 5′-TGA GAA GTG CTG ATG TAC CA- 3′			
*CXCL1*	for 5′-GAC CTT GCA GGG GAT TCA CCT C- 3′	Gärtner et al.^61^	125	60
	rev 5′-CGG GGT TGA GAC ACA CTT CCT G- 3′			
*CXCL3*	for 5'- GCC ATT GCC TGC AAA CTT -3'	Gärtner et al.^61^	189	56
	rev 5'- TGC TGC CCT TGT TTA GCA -3'			
*CXCL8*	for 5'-CGA TGC CAA TGC ATA AAA AC-3'	Danesh Mesgaran et al.^14^	153	56
	rev 5'-CTT TTC CTT GGG GTT TAG GC-3'			
*PTGS2*	for 5′-CTG TTG TCC CCA ACC AGG-3′	Odau et al.^38^	359	60
	rev 5′-CTG TCC AGC ACA GGC ATG-3′			
*MUC4*	for 5′-ACG TCA CTG TGC ATC TTT GG-3′	Danesh Mesgaran et al.^14^	199	60
	rev 5′-AAG CTC TTG ATG GAC GGT TG-3′			
*MUC6*	for 5′-CAG CAG TCC CAC TTC CTC TG-3′	Danesh Mesgaran et al.^14^	206	65
	rev 5′-CAG TGA TGG AGC TGG CTA GG-3′			
*MUC16*	for 5′-CAG GTC TCA AAA TCC CAT CC-3′	Danesh Mesgaran et al.^14^	256	62
	rev 5′-TGC TGG AGG TGT TGA TAT GG-3′			

Besides the genes of interest, qPCR was also performed for suppressor of zeste 12 homologue (SUZ12), Succinate dehydrogenase complex subunit A (SDHA), Ubiquitously eXpressed Transcript protein (UXT) and 60S ribosomal protein L19 gene (RPL-19), which serve as standardized reference genes for quantification. After controlling the reference genes for stability by geNorm^56^, RPL-19, SDHA and SUZ 12 were selected for the calculation of the normalization factor for the genes of interest (Table 3).

### Statistical analysis

Statistical analysis was performed in R version 4.0.2 (R Core Team (2020). Data was prepared for analysis using the package tidyverse v1.3.1^57^. We analysed the measured phenotypes (PMN percentage, *OVGP1*, *MUC4*, *MUC6*, *MUC16*, *IL1A*, *IL1B*, *PTGS2*, *CXCL1*, *CXCL3*, *CXCL8* and *HBDH*). Additionally, P4 concentration was included for calculation of customized residuals (see below). We found no differences in mRNA abundance between the ipsi- and contralateral oviduct or between day 0 and day 21 (both FOL), or day 10 and day 31 (both LUT) measured in heifers, respectively (Wilcoxon signed rank test with continuity correction; data not shown). Therefore, measurements from animals with two observations per day (one measure from ipsi- and contra-lateral oviduct each) were aggregated to the mean, and aggregated means of days 0, 21, 10 and 31 were analysed together, fitting univariate linear mixed models using the lmer function in the package lme4 v1.1-27^58^, via maximum likelihood estimation (option REML set to false).

Measured phenotypes were log10 transformed after adding a constant of one to every observation. The stage of the oestrous cycle was fitted as a fixed categorical effect with two levels, FOL and LUT, respectively. Animal was fitted as the random intercept to account for the covariance structure due to repeated measurements per individual. We also included a random intercept of day of experiment to reduce residual variance. Our data met all assumptions for linear mixed models. Residuals and random intercepts were normally distributed and residuals were homoscedastic.

Bar plots were created to visualize the results of our hypothesis testing based on back-transformed estimated marginal means (package emmeans v1.7.0, ggplot2 v3.3.5^59^, and ggpubr v0.4). In these plots the fitted model is shown as black dots, and whiskers represent the upper and lower 95% confidence intervals. P-values are from hypothesis testing of contrasts between estimated marginal means for the difference between the two oestrous stages, and were corrected for multiple testing across all phenotypes using the False Discovery Rate (FDR) approach proposed by Benjamini and Hochberg^60^. Significance was declared at a 5% false discovery rate (FDR).

Residuals, corrected for individual animal and day of experiment effects, were then calculated by subtracting the restricted maximum likelihood BLUP animal and day of experiment effects, (option REML set to true), estimated from the same linear mixed model as used for hypothesis testing, from the log10 transformed phenotypes after adding the constant of one.

Residuals were subjected to a Principal Component Analysis (package factoextra v1.0.7) using the prcomp function (option centre and scale set to true) and visualized via a biplot created with the fviz_pca_biplot function, which displays the PCA scores of samples (shown as dots) and loadings of each variable (shown as vectors) in the same graph. Dots closer together represent samples with similar values. The longer a vector of a variable the bigger the influence of said variable on that principal component. Vectors pointing in similar directions and forming small angles between them can be interpreted as positively correlated, vectors forming an angle of 90° as uncorrelated and vectors pointing in opposing directions as negatively correlated.

The heatmap was drawn (package and function pheatmap v1.0.12) using the “ward.D2” method for hierarchical clustering, based on correlation coefficients as distance measures for rows and columns. Residuals were centred and scaled (z-transformed) within each phenotype across all samples.

The correlation plot, also based on residuals, calculated as described above, was produced with the package corrplot v0.92. Phenotypes were clustered using the “ward.D2” method. Each cell displays the estimated Pearson correlation coefficient. If a correlation coefficient is significantly different from zero, after multiple testing correction at 5% FDR across all estimated correlation coefficients, a heatmap like a coloured circle, with a circumference representing the size of the estimated correlation coefficient, is added to a cell.

## Data Availability

All data generated or analysed during this study are included in this article and its supplementary information files. The datasets generated and/or analysed during the current study are available from the corresponding author on reasonable request.
